# Schools’ Readiness for Child Sexual Abuse Prevention Education: an Overview of Theoretical Models

**DOI:** 10.1007/s11121-025-01843-6

**Published:** 2025-10-01

**Authors:** Yuejiao Wu, Kerryann Walsh, Sonia L. J. White, Lyra L’Estrange

**Affiliations:** School of Education, Faculty of Creative Industries, Education and Social Justice, Queensland University of Technology, Victoria Park Rd, Kelvin Grove, Brisbane, QLD 4059 Australia

**Keywords:** Readiness, Measure, School violence, Child sexual abuse, Prevention

## Abstract

Schools’ readiness appears an important factor influencing their implementation of violence prevention programs. This review was undertaken to identify, describe, and compare existing theoretical readiness models and their strengths and limitations, and to select an appropriate theoretical model to underpin the study of schools’ readiness for child sexual abuse (CSA) prevention education. This, in turn, would guide development of a new instrument to assess schools’ readiness for CSA prevention. Searches were conducted from September to December 2022 in ERIC, PsychINFO, PubMed, Science Direct, Sociological Abstracts, Web of Science, and Google Scholar, and handsearches were made in academic journals. We included peer-reviewed papers published in English that reported the development, testing, or use of a theoretical readiness model at an organizational level. We identified three candidate groups of theoretical models from 85 papers: the community readiness model, the multidimensional child maltreatment prevention readiness model, and organizational readiness for change theories. These models were appraised using four criteria for selecting implementation science theories and frameworks (Birken et al. 2017). We propose Weiner’s (2009) organizational readiness for change as the most plausible theoretical model with both descriptive and analytical potential for assessing schools’ readiness for child sexual abuse prevention education, and discuss the conceptual and empirical strengths and weaknesses of the identified models. The review has demonstrated the utility of applying criteria (Birken et al. 2017) to appraise and select theoretical readiness models in CSA prevention education and other implementation research areas.

## Background

Child sexual abuse (CSA) is a global public health problem, which can endanger children’s short- and long-term health and wellbeing. It is defined as “the involvement of a child in sexual activity that he or she does not fully comprehend, is unable to give informed consent to, or for which the child is not developmentally prepared and cannot give consent, or that violate the laws or social taboos of society. Children can be sexually abused by both adults and other children who are—by virtue of their age or stage of development—in a position of responsibility, trust or power over the victim” (World Health Organization, 2006, p. 10). CSA can lead to range of physical, mental, and behavioral problems (Harris et al., 2024; Hughes et al., 2017; Lawrence et al., 2023), such as cardiac and gastrointestinal illnesses, obesity, depression, anxiety, self-harm, suicide ideation, sexual risk behaviors, drug and alcohol misuse, smoking (e.g., Hailes et al., 2019; Maniglio, 2013; McCarthy-Jones & McCarthy-Jones, 2014; Ullman, 2016). Additionally, CSA can adversely effect interpersonal relationships (Marson, 2024) and academic outcomes (Fry et al., 2018). CSA is also acknowledged to create economic burdens at the social level (Letourneau et al., 2018).

Education is a key strategy in the primary prevention of violence against children in general (World Health Organization, 2018) and CSA in particular (World Health Organization, 2019). Schools have been seen as an appropriate context in which to teach CSA prevention education because of their ability to reach a large number of children in a relatively cost-efficient way—recently estimated in the range of $34–64 USD per student (Shipe et al., 2022). The key elements of CSA prevention education can be aligned with school curricula (Walsh et al., 2013), and prevention education can also ripple out, via children, to parents and communities (Devries & Naker, 2021; Nickerson et al., 2021). In addition, schools have readily available buildings, equipment, and personnel that can be used at a practical level to deliver CSA prevention education (Russell et al., 2020).

Child sexual abuse prevention education in schools comprises initiatives (e.g., curricula, programs, resources) implemented into school settings by teachers, aiming to increase children’s knowledge and skills with the ultimate goal of reducing CSA. Important to CSA prevention education is the schools’ readiness to implement the evidence-based programs. Schools’ readiness (i.e., how prepared and well-equipped they are) can influence whether or not schools choose to teach CSA prevention education and the degree to which they cover evidence-based content. Readiness is a multidimensional construct simultaneously comprising processes, contexts, and individuals. It reflects the extent to which individuals (such as teachers) or collections of individuals (such as a school community) are inclined to “accept, embrace, and adopt a particular plan to purposefully alter the status quo” (Holt et al., 2007a, 2007b, p. 326).

**Fig. 1:**
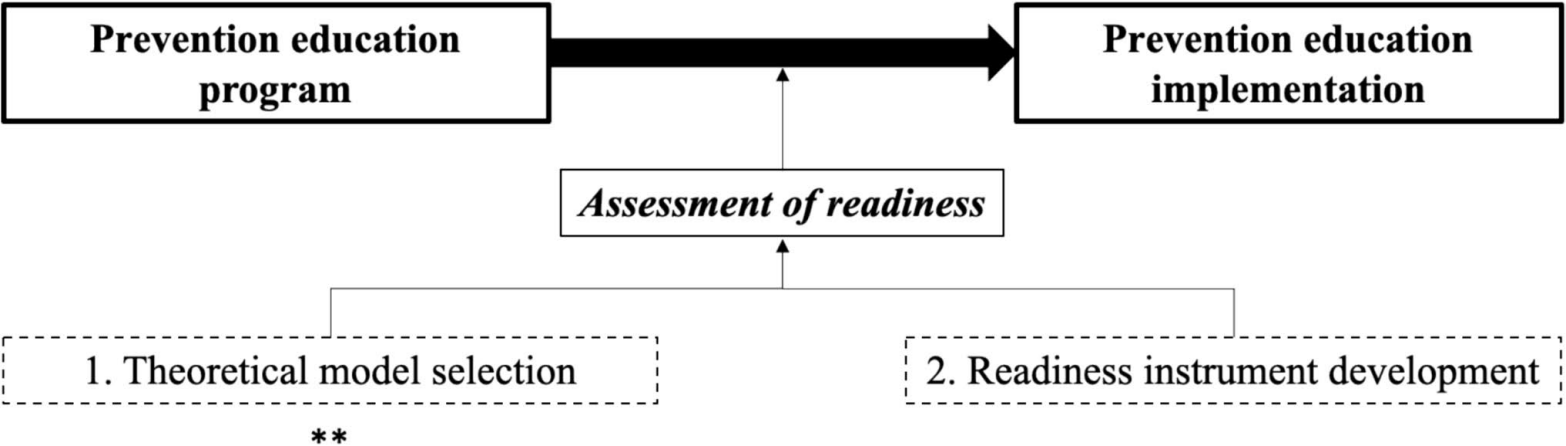
Conceptualizing the importance of assessing readiness prior to implementing prevention education programs. Note. **: This is the central focus of this study

The assessment of readiness is considered a critical precursor for implementation of prevention education programs. Importantly, the assessment of readiness needs to be specific to the contents of the program and the context in which it will be implemented (Fig. 1). While schools’ readiness for nutritional intervention (Octaria et al., 2021), school reform (Galdames et al., 2017), suicide prevention (Breux & Boccio, 2019), and curriculum change (Jippes et al., 2013) have been previously studied, schools’ readiness for CSA prevention education has not been systematically studied to date. To develop a new instrument to assess schools’ readiness for CSA prevention education, the first step is to select a theoretical model for the content and the context.

The aim of this overview is to identify, describe, and compare existing theoretical readiness models and their strengths and limitations, and to select an appropriate theoretical model to underpin the study of schools’ readiness for CSA prevention education and, in turn, to guide development of a new instrument to assess schools’ readiness for CSA prevention education. For the purpose of this overview, a theoretical model will be defined, as it is used in implementation science, as “a set of interrelated concepts, definitions, and propositions that present a systematic view of events or situations by specifying relations among variables, in order to explain and predict events or situations” (Glanz et al., 2015, p. 26). Theoretical models provide a synthesizing architecture for implementation science which may offer a more efficient and appropriate approach to generalize findings across various settings within implementation science (Birken et al., 2017). Theoretical models guide implementation, help to identify key determinants of implementation success, and the selection of appropriate strategies. They also assist in framing study questions, motivating hypotheses, anchoring background literature, clarifying constructs for measurement, illustrating relationships to be tested, and contextualizing results (Proctor et al., 2012).

## Method

We classify this study as an “overview” according to Grant and Booth’s (2009, p. 94) typology of systematic and non-systematic reviews. An overview attempts to summarize and characterize the extant literature. It may or may not include systematic searching and typically involves narrative synthesis. We began this overview (Grant & Booth, 2009) of theoretical models by designing a systematic search strategy based on PRISMA search extension guidelines (Rethlefsen et al., 2021) to identify peer-reviewed empirical studies. Six electronic databases (ERIC, PsychINFO, PubMed, Science Direct, Sociological Abstracts, Web of Science), and one web search engine indexing metadata of scholarly literature (Google Scholar) were searched using combinations of the terms: “child sexual abuse,” “prevention,” “education,” “school*,” and “readiness.” No date limiters were applied. The search was conducted from September to December 2022. Duplicates were removed and records were screened for eligibility by a single author. No relevant records were retrieved (Table 1).

**Table 1:** Search strategy: databases, search terms, and results

Search	Database	Searching terms	Results
1	EBSCOhost (ERIC, PsychINFO)	“child sexual abuse” AND “prevention” AND “education” AND “school” AND “readiness”	8 None relevant
1	PubMed	“child sexual abuse” AND “prevention” AND “education” AND “school” AND “readiness”	8 None relevant
1	Web of Science	“child sexual abuse” AND “prevention” AND “education” AND “school” AND “readiness”	7 None relevant
1	Science Direct	“child sexual abuse” AND “prevention” AND “education” AND “school” AND “readiness”	106 None relevant
1	Sociological Abstracts	“child sexual abuse” AND “prevention” AND “education” AND “school” AND “readiness”	1413 None relevant
1	Google Scholar	“child sexual abuse” AND “prevention” AND “education” AND “school” AND “readiness”	6030 None relevant

As no relevant records were identified from systematic searching, we applied a sequence of non-systematic “supplementary search techniques” (Cooper et al., 2017, p. 1) in which search strings were refined to include only “readiness” and “prevention.” To maintain the focus on the research field of child protection, we used handsearching of highly relevant academic journals (*Child Abuse & Neglect*, Child Maltreatment, *Journal of Child Sexual Abuse*) to identify potentially relevant studies and clusters of studies. Then, we used the Google Scholar advanced search function searching for all of the words “readiness” and “prevention” to identify potentially relevant studies published in other peer-reviewed journals published in English. The first 200 titles and descriptors appearing in the search results were screened following the recommendations of Haddaway et al. (2015). Again, no date limiters were applied.

Records from academic database and Google Scholar searches were combined and screened for duplicates. The full-text articles of remaining records were retrieved and assessed for eligibility. Full-text articles with theoretical models about organizational readiness were included for further analysis. The theoretical models in each article were described and compared to select the most appropriate theoretical model for developing an instrument to assess schools’ readiness for CSA prevention education. To determine the strengths and limitations of the theoretical models, Birken’s appraisal and selection criteria were adapted and applied (Birken et al., 2017).

With a focus on schools’ readiness to implement CSA prevention education programs, four priority criteria from Birken et al. (2017) were used to appraise and select theoretical models: (i) analytic level (operationalized to refer to the theory’s proposed application level, e.g., individual or organizational level); (ii) generalizability (operationalized as the theory’s applicability to CSA prevention); (iii) application to a specific setting or population (operationalized as restricted to schools and teachers/school staff); and (iv) associated research method (operationalized as the potential for the model to act as a foundation for developing a questionnaire to assess schools’ readiness).

## Results

**Fig. 2:**
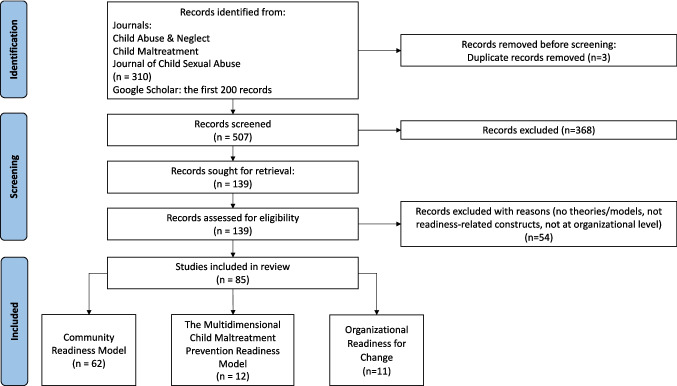
Study screening and selection process

An abridged PRISMA flowchart is shown in Fig. 2. After removing duplicates and irrelevant records, 139 records were screened against inclusion criteria. Theoretical models were identified in 85 papers published from 1995 to 2022. Each study was thematically classified according to the underpinning theory or model identified in the paper.

We identified three candidate models. These comprised two specific theoretical models: the community readiness model (62 papers) (Oetting et al., 1995) and the multidimensional model for child maltreatment prevention readiness in low- and middle-income countries (12 papers) (Mikton et al., 2011), and one “family” of theoretical models known as “organizational readiness for change” (11 papers) (Weiner et al., 2008, 2020). A description on each of these theoretical readiness models will be presented in turn, followed by a summary of the strengths and limitations, and appropriate model selection.

### Community Readiness Model

The community readiness model (*n* = 62 papers) was originally developed by researchers at the Tri-Ethic Center for Prevention Research, Colorado State University, USA, to assess rural communities’ readiness for engaging in the prevention of high-risk drug and alcohol use (Oetting et al., 1995). In turn, this model was based on theories and concepts from psychological readiness (Prochaska et al., 1994), diffusion of innovations (Rogers, 1983), and community development (Oetting et al., 1995). A recent systematic review tracing the scope and use of community readiness tools found 40 studies spanning 14 diverse social and health issues (Kostadinov et al., 2015), including drug and alcohol abuse (Plested et al., 1999), intimate partner violence (Turell et al., 2012), obesity prevention (Plested et al., 1999), HIV/AIDS (Thurman et al., 2007), brain injury (Stallones et al., 2008), teen pregnancy prevention (Bhuiya et al., 2017), COVID-19 prevention (Adane et al., 2021), nutrition intervention (Octaria et al., 2021), and sexuality education (Ahmed et al., 2021). The Community Readiness Tool has five dimensions: (i) community knowledge of efforts, (ii) community climate, (iii) leadership, (iv) knowledge of prevention, and (v) resources. Data are typically collected in interviews with a small number of key informants who respond to open-ended questions. Researchers score informant responses on each of the five dimensions using an anchored 9-point rating scale. Overall community readiness is calculated as the mean of the five dimensions’ scores.

#### The Multidimensional Child Maltreatment Prevention Readiness Model

The multidimensional child maltreatment prevention readiness model (*n* = 12 papers) was developed by the World Health Organization (WHO) (Mikton et al., 2011) to assess child maltreatment prevention readiness at national, subnational, and community levels in low- and middle-income countries. It was developed as part of a broader program of work aimed at assessing prevalence rates for child maltreatment and planning interventions for child maltreatment in low- and middle-income countries (World Health Organization, 2013). The WHO developed this model in three stages involving conceptual review, expert consultation, and country focus groups. The WHO reviewed 28 models of community readiness and community capacity and researchers extracted statements pertaining to eight readiness dimensions (problem assessment; mobilization to address the problem; human and technical resources; material resources; institutional resources and linkages; informal social resources; legislation, policies, and plans; and programs). After expert consultation and country focus groups, the WHO model resulted in 10 readiness dimensions: (i) key country conditions; (ii) problem assessment; (iii) legislation, mandates, policies, and plans; (iv) willingness to address the problem; (v) institutional links and intersectoral collaboration; (vi) institutional resources and efficiency; (vii) material resources; (viii) human and technical resources; (ix) informal social resources; and (x) program implementation and evaluation. Based on this model, WHO employed four further instrument development stages, including cognitive testing, pilot testing, field testing, and final revision of the instrument after field testing. The Readiness Assessment for the Prevention of Child Maltreatment (RAP-CM) tool can assess a country’s readiness to implement evidence-based child maltreatment prevention programs on a large scale.

Using the RAP-CM tool, Mikton and colleagues (2013) assessed child maltreatment prevention readiness in five countries: Brazil, the Former Yugoslav, Republic of Macedonia, Malaysia, Saudi Arabia, and South Africa. Later, Al Saadoon et al. (2020) assessed child maltreatment prevention readiness in Oman. The RAP-CM has also been used to assess readiness for child maltreatment prevention at the community level (Gagne et al., 2020). It has recently begun to be adapted for use in different fields, for instance, in alcohol-related harm prevention (Swahn et al., 2022).

### Organizational Readiness for Change Theories

The family of theoretical models with the longest history are the readiness for change theories (*n* = 11 papers). Lewin (1947) first proposed that individuals progress through change in three stages: unfreezing, moving, and refreezing. Based on this broad conception of individual level change, researchers have since worked to understand change at an organizational level (Armenakis et al., 2007). Today, there exist conceptions of both individual readiness for change and organizational readiness for change. Individual readiness for change is sometimes viewed as a part of organizational readiness for change (Backer, 1995). As the current overview focuses on theoretical readiness models at an organizational level to assess schools’ readiness for CSA prevention education, only organizational readiness for change (ORC) was included.

Organizational readiness for change (ORC) (*n* = 11 papers) is considered a critical precursor to successful implementation. It has been suggested that as many as half of all implementation initiatives fail because of inadequate preparation for organizational change (Weiner et al., 2020). A case in point is tobacco cessation services (one of the most cost-effective healthcare interventions in the USA). However, research had identified significant implementation problems (Bernstein et al., 2013; Rojewski et al., 2019). One consistent finding in these studies is that healthcare services have not fully realized the importance of readiness for change (Holt et al., 2010). Organizational readiness for change has very wide applicability and flexibility (Weiner et al., 2008, 2020), and it has been applied in various fields including in business (e.g., Dwayne Simpson, 2009), education (e.g., Bank et al., 2017; Chavarkar et al., 2021), healthcare (e.g., Al-Hussami et al., 2018; Carlsson & Wadensten, 2018), and child welfare (Winters et al., 2020).

Organizational readiness for change is a multi-level and multi-faceted construct which can be assessed at individual and/or organizational level (Weiner et al., 2020). There is no single agreed definition of organizational readiness for change and, in the research literature, studies have defined it in one of several ways. Some studies have used a psychological definition (Armenakis et al., 1993), yet others have used a psychological and structural definition (Holt 2016; Weiner, 2009). Psychological components include personal factors such as feelings and opinions about change (e.g., Jones et al. 2005; Bouckenooghe 2010). Structural components are broader conditions within organizations such as organizational capacity, climate, or innovation (Holt et al., 2010; Scaccia et al., 2015). Measurement of organizational readiness for change, therefore, depends on how readiness is conceptualized and defined.

Research on organizational readiness for change has been synthesized in several comprehensive reviews, including reviews of its measurement (Gagnon et al., 2014; Weiner et al., 2008, 2020). The four most frequently used measures of organizational readiness for change include the Organizational Change Recipients’ Beliefs Scale (Armenakis et al., 2007), the Organizational Readiness to Change Assessment (Helfrich et al., 2009), the Individual Readiness for Organizational Change (Holt et al., 2007a, 2007b), and Texas Christian University Organizational Readiness for Change (Lehman et al., 2002). Each enables measurement at individual and/or organizational level. However, in a critical review, Weiner et al. (2020) found that not all of these four measures were equally sound. For example, the Organizational Readiness to Change Assessment (Helfrich et al., 2009), used mainly in health settings, and the Texas Christian University Organizational Readiness for change (Lehman et al., 2002), used mainly in substance misuse and mental health settings, have reported limited reliability and validity (Weiner et al., 2020).

Organizational readiness for change theories including Weiner’s (2009) and Holt’s (2016) theoretical models have been used in educational settings to develop change measurement scales (Jippes et al., 2013; Kondakçı & Zayim Kurtay, 2013) or frameworks (Sharma et al., 2014). Schools’ readiness for change on a diverse array of topics has been studied (Budur et al., 2022; Hjort et al., 2021; Hustus & Owens, 2018; Ittner et al., 2019; Zakaria & Ismail, 2020). Study participants have been teachers (Budur et al., 2022; Hjort et al., 2021), principals (Ittner et al., 2019), and administrators (Hustus & Owens, 2018) working in primary (elementary) schools, secondary (high) schools, colleges, and universities. Theoretical model constructs have been measured via quantitative (Zakaria & Ismail, 2020) or qualitative (Hjort et al., 2021) methods using existing, adapted, and custom-made instruments, the psychometric properties of which have been inconsistently reported or lacking.

Both Holt et al.’s (2010) and Weiner’s (2009) organizational readiness for change theory have been widely used in different fields to assess readiness including educational settings. However, Holt et al.’s (2010) theory has been more applied at the individual level than organizational level. Weiner’s (2009) organizational readiness for change theory has been developed for assessing readiness at the organizational level with a validated scale in health setting. Therefore, Weiner’s (2009) ORC theory was considered the representative theory from organizational readiness for change theories.

### Strengths and Limitations of Theoretical Models and Appropriate Theoretical Model Selection

The descriptions above detail the strengths and limitations of each of the identified theoretical models. These strengths and limitations are summarized in Table 2.

**Table 2:** Strengths and limitations of identified theoretical models

Theoretical model	Strengths	Limitations
Community readiness model	• Focuses on whole-of-community readiness • Can be used to create community-specific and culturally-specific interventions (Tri-Ethic Center for Prevention Research 2014)	• Requires substantial time and resource commitment to complete assessment • Use of subjective scoring by researchers • Potential for social desirability bias • Changing and transient nature of readiness is unsuited to measurement at a single point in time • Limited power to statistically detect significant findings given relatively few interviews per community, and few communities • No specific application to CSA prevention field (Kostadinov et al., 2015)
The multidimensional child maltreatment prevention readiness model	• Developed in a rigorous multi-stage process conducted in six countries • Best suited to assessing readiness for child maltreatment prevention at the country level, region, or community level as it is concerned with prevention program implementation on a large scale • Clear and detailed handbook for researchers to use • Technical reports on the instrument development are publicly available • Data can be compared with responses from researchers, experts, and clinicians via the RAP-CM-XD (arguably a more objective assessment) (Mikton et al. 2013; Swahn et al., 2022; World Health Organization, 2013)	• Not derived from established theory • Validity and reliability can be determined only after administration • Assessment is lengthy and can be completed only by individuals with very in-depth knowledge of intra-country child maltreatment governance and prevention program implementation • Assessment tool is designed to assess readiness for child maltreatment prevention broadly (meaning physical abuse, emotional abuse, sexual abuse, and neglect) • No specific application to CSA prevention field (Guo et al. 2019; Zhang et al. 2015; Mikton et al., 2011)
Weiner’s organizational readiness for change	• Comprehensive • Focuses on organizational level change • Can be used in various different contexts • Several existing instruments • Used in education settings (Shea et al., 2014; Weiner, 2009; Weiner et al., 2020)	• No single tool available • Existing instruments lack validity and reliability • No specific application to CSA prevention field (Weiner, 2009; Weiner et al., 2020)

As mentioned in the “Method” section, the four criteria from Birken et al. (2017) were applied to inform theoretical readiness model selection: analytic level, generalizability, application to settings, and associated research method. These four criteria aligned with the aims of the current study, as we wanted this theoretical model to be generalizable and applicable to various settings with the potential to be developed into a scale to measure schools’ readiness for CSA prevention education at the organizational level. Moreover, these four criteria have been considered important and commonly chosen when the implementation scientists select theories (Birken et al., 2017). Table 3 shows a comparison of theoretical models against these four criteria. Weiner’s organizational readiness for change theory met all four criteria. It is an organizational level theory which could be used in different settings with an existing validated Organizational Readiness for Implementing Change (ORIC) scale (Shea et al., 2014) that may be used or adapted for survey administration. There are empirical studies that have already applied this theory and the ORIC scale to assess organizational readiness (Esan et al., 2022; Faris et al., 2023; Swindle et al., 2018). Consequently, Weiner’s organizational readiness for change theory was selected to assess schools’ readiness for CSA prevention education.

**Table 3:** Comparison of theoretical models (criteria adapted from Birken et al., 2017)

Criteria	Community readiness model	The multidimensional child maltreatment prevention readiness model	Weiner’s organizational readiness for change theory
Analytical level: organizational and individual	✔	✔	✔
Generalizability: applicability to various disciplines, settings, and populations	✘	✘	✔
Application: relevance to specific setting or population	✔	✔	✔
Associated research method: can be used to develop a valid questionnaire or methodology for constructing one	✔	✔	✔

### Weiner’s Organizational Readiness for Change Theory

**Fig. 3:**
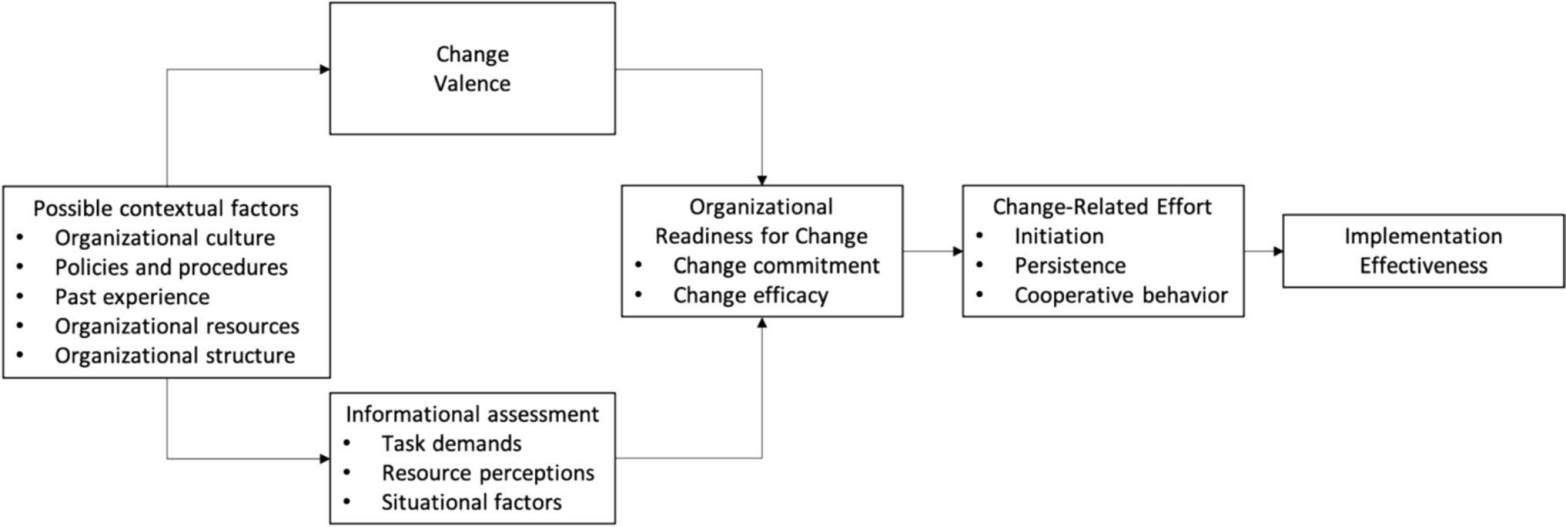
Determinants and outcomes of organizational readiness for change (Weiner, 2009, p.4)

Weiner’s (2009) organizational readiness for change (ORC) theory is one of the most widely used ORC theories. Weiner defined ORC as the “extent to which organizational members are psychologically and behaviorally prepared to implement change” (Weiner, 2009, p. 2). Weiner defined ORC comprehensively according to both psychological and structural dimensions at an organizational level. The theory provides a means of reconciling claims that structural factors shape the readiness perception (Weiner, 2009). It enables the integration of psychological and structural perspectives on organizational readiness for change. This seems fit for purpose in that our current research aims to investigate schools’ readiness for CSA prevention education and to find a way to assess this. Figure 3 shows the conceptual model for Weiner’s ORC theory (Weiner, 2009, p. 4). It shows that ORC is a two-faceted construct influenced by various conditions. The two facets are change commitment and change efficacy. Contextual factors is a broad condition to affect ORC. Change valence is a determinant of change commitment, as well as informational assessment is a determinant of change efficacy.

## Discussion

In this review, we aimed to identify, describe, and compare existing theoretical readiness models and their strengths and limitations, and to select an appropriate theoretical model to assess schools’ readiness for CSA prevention education. The application of Birken et al.’s (2017) criteria for theory selection allowed for specific alignment with the CSA prevention education content and intended school context. Two specific models, the community readiness model (CRM) (Oetting et al., 1995) and the multidimensional model for child maltreatment prevention readiness in low- and middle-income countries (Mikton et al., 2011), were identified, and one family of theoretical models, organizational readiness for change (Weiner et al., 2008, 2020), were found. Based on their strengths and limitations and analysis against four key implementation quality criteria, Weiner’s (2009) organizational readiness for change (ORC) theory emerged as the strongest candidate for adoption. This process also offers a transferable approach for theory selection in readiness research across other contexts. We now discuss this theoretical model, its existing applications, and potential for application in future research.

### Weiner’s Organizational Readiness for Change Theory

As stated previously, Weiner defined ORC as the “extent to which organizational members are psychologically and behaviorally prepared to implement change” (Weiner, 2009, p. 2). The next sections discuss the constructs of contextual factors, change valence, informational assessment, and organizational readiness for change as they might relate to school-based program implementation, including CSA prevention education programs.

### Contextual Factors

In Weiner’s theory, contextual conditions affect organizational readiness through more proximal conditions. Organizational culture, policies and procedures, past experiences with change, organizational resources, and structures (such as funding and governance) could positively or negatively affect members’ assessment of task demands, resource perception, and situational factors and change valence (Weiner, 2009). For CSA prevention education, contextual factors include school climate and culture, relevant child protection policies, and communication networks with families, all of which shape schools’ capacity for prevention (Orchowski et al., 2023; Payne & Eckert, 2010; Thaker et al., 2008).

### Change Valence

Change valence is the extent to which organizational members value the change. For instance, do they think that is needed, important, beneficial, or worthwhile? The key point is whether members value the change enough to commit to implementing the change. For CSA prevention education, change valence refers to the attitudes, beliefs, perceptions of school teachers toward such education, reflecting the degree to which they value its implementation in schools.

### Informational Assessment

Informational assessment comprises organizational members’ perceptions of task demands, resources perceptions, and situational factors. The “task demands” means the task knowledge, understanding what and how to implement, whereas “resources perceptions” assesses the quality and availability of resources. In the context of CSA prevention education, resource perception relates to the human, financial, material, time resources required for implementation. The “situational factors” are the given circumstances that organizational members currently face, such as favorable or unfavorable timing (Shea et al., 2014; Weiner, 2009). In school contexts, situational factors are associated with perspectives toward support of teachers, schools, families, and communities regarding the implementation of CSA prevention.

### Organizational Readiness for Change

There are two facets of organizational readiness for change. The first facet is change commitment, which reflects organizational members’ shared resolve to implement a change. In the context of CSA prevention education, change commitment refers to the collective willingness of school teachers and leadership to prioritize and take action to embed CSA prevention education in schools. The second facet is change efficacy, which reflects members’ shared belief in their collective capability to implement a change. It is a type of collective efficacy, a construct derived from social cognitive theory. It is a comprehensive summary or judgment of perceived capability for performing a special task (Bandura, 1977; Gist & Mitchell, 1992). When members share common and favorable assessment of these determinants of implementation capability, their change efficacy is high (Weiner, 2009). In context of CSA prevention education, change efficacy refers to the collective of school teachers’ confidence and capability to implement CSA prevention education.

### Utility of Weiner’s Theory for Instrument Development

Weiner’s ORC theory has been used widely to assess readiness in different research fields, such as health care (Adamu et al., 2020; Adelson et al., 2021; Akande et al., 2019), mental health (Hannon et al., 2012), child welfare (Winters et al., 2020), tobacco use (Le et al., 2021; Lidegaard et al., 2021), and education (Arthur et al., 2020; Jippes et al., 2013). Some researchers have developed customized instruments for assessing organizational readiness based on Weiner’s ORC theory (e.g., Jippes et al., 2013; Oostendorp et al., 2015), whereas others have adapted and applied an existing instrument. The most commonly adapted of these is known as organizational readiness for change (ORIC) (Shea et al., 2014).

Based on Weiner’s theory, Shea et al. (2014) originally developed the ORIC for use in healthcare settings as a theory-based psychometrically validated measurement tool that has since been adapted and applied in several studies both within and outside healthcare settings (e.g., Adamu et al., 2020; Adelson et al., 2021; Winters et al., 2020).

It is thus possible for researchers to develop or adapt theory-based scales for specific readiness issues or topics and to modify these scales to fit their studies, as the various studies above have done. The successful applications of Weiner’s ORC theory to the development of new instruments in various disciplinary areas provides strong support for its use in developing an instrument to assess schools’ readiness for CSA prevention education.

### Implication for Future Research

The current study provides important implications. First, this study demonstrates the utility of Weiner’s ORC theory as a conceptual foundation for assessing schools’ readiness for implementing CSA prevention education. Four main constructs (contextual factors, informational assessment, change valence, and organizational readiness for change) offer a theoretically grounded structure from which scales can be developed. Contextual factors, informational assessment, and change valence could be used to identify and measure the potential school-level influencing factors for schools’ readiness. Organizational readiness for change could be applied to assess schools’ readiness as a desired outcome. Further, the application of this theory in the CSA prevention education context could also suggest its broader relevance for readiness assessments in other areas of child protection and school-based prevention.

Second, the current study contributes a transparent and replicable theoretical model selection method for implementation research. By explicitly aligning theory selection with study aims, prevention characteristics, and instrument needs, this approach enhances conceptual clarity and empirical coherence. Researchers in implementation science may adapt this model selection process to guide their theoretical decisions in the future to strengthen the theoretical underpinnings of implementation research and practice.

### Study Limitations

The key limitation of this overview lies in the combined use of systematic and non-systematic search strategies. On the one hand, these approaches can be considered complementary. The systematic search component yielded no relevant records. The non-systematic search component enabled us to purposively pursue relevant papers unencumbered by the restrictions of a narrow search frame. On the other hand, it offers less security that we have captured all relevant work—it is plausible that some may have been missed. Our appraisal of the theoretical models, however, reflects a rigorous approach to advancing understanding of the contributions of each reviewed model and justifies our selection of one particular theory over others.

## Conclusion

This review aimed to identify, describe, and compare existing theoretical readiness models and their strengths and limitations, and to select an appropriate theoretical model to underpin the study of schools’ readiness for CSA prevention education and to develop a new instrument to assess schools’ readiness for CSA prevention. Three categories of theoretical models were identified in the literature review to assess readiness at organizational level in various research areas which could be referred to by other researchers in the future readiness studies. After examining several features of these theoretical models according to Birken et al. (2017), we concluded and recommended that Weiner’s organization readiness for change theory offered the greatest promise and flexibility for researching schools’ readiness for CSA prevention education.

## References

[CR1] Adamu, A. A., Gadanya, M. A., Jalo, R. I., Uthman, O. A., Nnaji, C. A., Bello, I. W., & Wiysonge, C. S. (2020). Assessing readiness to implement routine immunization among patent and proprietary medicine vendors in Kano, Nigeria: A theory-informed cross-sectional study. *Expert Review of Vaccines,**19*(4), 395–405. 10.1080/14760584.2020.1750379 32238070 10.1080/14760584.2020.1750379

[CR2] Adane, D., Yeshaneh, A., Wassihun, B., & Gasheneit, A. (2021). Level of community readiness for the prevention of covid-19 pandemic and associated factors among residents of Awi zone, Ethiopia: A community-based cross-sectional study. *Risk Management And Healthcare Policy,**14*, 1509–1524. 10.2147/RMHP.S302974 33883957 10.2147/RMHP.S302974 PMC8053606

[CR3] Adelson, P., Yates, R., Fleet, J. A., & McKellar, L. (2021). Measuring organizational readiness for implementing change (ORIC) in a new midwifery model of care in rural South Australia. *BMC Health Services Research,**21*(1), Article 368. 10.1186/s12913-021-06373-9 33879145 10.1186/s12913-021-06373-9 PMC8056551

[CR4] Ahmed, F., Ahmad, G., Paff, K., Samkange-Zeeb, F., & Brand, T. (2021). A cross-sectional community readiness assessment for implementing school-based comprehensive sexuality education in Islamabad, Pakistan. *International Journal of Environmental Research and Public Health*. 10.3390/ijerph18041497 33557441 10.3390/ijerph18041497 PMC7914735

[CR5] Akande, V. O., Ruiter, R. A. C., & Kremers, S. P. J. (2019). Exploring Nunavut public health system’s readiness to implement obesity prevention policies and programs in the Canadian Arctic. *BioMed Research International,**2019*, Article 1584956. 10.1155/2019/1584956 31211133 10.1155/2019/1584956 PMC6532304

[CR6] Al Saadoon, M., Al Numani, A., Saleheen, H., Almuneef, M., & Al-Eissa, M. (2020). Child maltreatment prevention readiness assessment in Oman. *Sultan Qaboos University Medical Journal*, *20*(1), e37-e44. 10.18295/squmj.2020.20.01.006 10.18295/squmj.2020.20.01.006 PMC7065698 32190368

[CR7] Al-Hussami, M., Hammad, S., & Alsoleihat, F. (2018). The influence of leadership behavior, organizational commitment, organizational support, subjective career success on organizational readiness for change in healthcare organizations. *Leadership in Health Services,**31*(4), 354–370. 10.1108/LHS-06-2017-0031 30234452 10.1108/LHS-06-2017-0031

[CR8] Armenakis, A. A., Bernerth, J. B., Pitts, J. P., & Walker, H. J. (2007). Organizational change recipients’ beliefs scale: Development of an assessment instrument. *The Journal of Applied Behavioral Science,**43*(4), 481–505. 10.1177/0021886307303654

[CR9] Armenakis, A. A., Harris, S. G., & Mossholder, K. W. (1993). Creating readiness for organizational change. *Human Relations,**46*(6), 681–703. 10.1177/001872679304600601

[CR10] Arthur, K., Christofides, N., & Nelson, G. (2020). Educators’ perceptions of organisational readiness for implementation of a pre-adolescent transdisciplinary school health intervention for inter-generational outcomes. *PLoS One,**15*(1), Article e0227519. 10.1371/journal.pone.0227519 31914148 10.1371/journal.pone.0227519 PMC6948754

[CR11] Backer, T. E. (1995). Assessing and enhancing readiness for change: Implications for technology transfer. *NIDA Research Monograph,**155*, 21–41. 8594459

[CR12] Bandura, A. (1977). Self-efficacy: Toward a unifying theory of behavioral change. *Psychological Review,**84*(2), 191–215. 10.1037/0033-295x.84.2.191 847061 10.1037//0033-295x.84.2.191

[CR13] Bank, L., Jippes, M., Leppink, J., Scherpbier, A. J., den Rooyen, C., van Luijk, S. J., & Scheele, F. (2017). Are they ready? Organizational readiness for change among clinical teaching teams. *Advances in Medical Education and Practice,**8*, 807–815. 10.2147/AMEP.S146021 29276424 10.2147/AMEP.S146021 PMC5733925

[CR14] Bernstein, S. L., Yu, S., Post, L. A., Dziura, J., & Rigotti, N. A. (2013). Undertreatment of tobacco use relative to other chronic conditions. *American Journal of Public Health,**103*(8), e59–e65. 10.2105/AJPH.2012.301112 23763395 10.2105/AJPH.2012.301112 PMC4007856

[CR15] Bhuiya, N. H., L. Duane, Desmarais, J. F., Erica , Conlin, M., Perez-McAdoo, S., Waggett, J., & Tendulkar, S. A. (2017). Strategies to build readiness in community mobilization efforts for implementation in a multi-year teen pregnancy prevention initiative. *Journal of Adolescent Health*, *60*(3), S51-S56. 10.1016/j.jadohealth.2016.11.001 10.1016/j.jadohealth.2016.11.001 PMC6518403 28235436

[CR16] Birken, S. A., Powell, B. J., Shea, C. M., Haines, E. R., Alexis Kirk, M., Leeman, J., Rohweder, C., Damschroder, L., & Presseau, J. (2017). Criteria for selecting implementation science theories and frameworks: Results from an international survey. *Implementation Science,**12*(1), 124. 10.1186/s13012-017-0656-y 29084566 10.1186/s13012-017-0656-y PMC5663064

[CR17] Bouckenooghe, D. (2010). Positioning change recipients’ attitudes toward change in the organizational change literature. *The Journal of Applied Behavioral Science*, *46*(4), 500-531. 10.1177/0021886310367944

[CR18] Breux, P., & Boccio, D. E. (2019). Improving schools’ readiness for involvement in suicide prevention: An evaluation of the Creating Suicide Safety in Schools (CSSS) workshop. *International Journal of Environmental Research and Public Health,**16*(12), Article 2165. 10.3390/ijerph16122165 31248082 10.3390/ijerph16122165 PMC6617090

[CR19] Budur, T., Demir, A., & Cura, F. (2022). University readiness to online education during Covid-19 pandemic. *International Journal of Social Sciences & Educational Studies*,* 8*(1), 180–200. 10.23918/ijsses.v8i1p180

[CR20] Carlsson, O. U., & Wadensten, B. (2018). Professional practice-related training and organizational readiness for change facilitate implementation of projects on the national core value system in care of older people. *Nursing Open,**5*(4), 593–600. 10.1002/nop2.185 30338105 10.1002/nop2.185 PMC6177551

[CR21] Chavarkar, M. G., Hultgren, M., & Lommel, L. (2021). Revising an organizational readiness tool for doctor of nursing practice projects. *Nurse Educator,**46*(3), 170–173. 10.1097/NNE.0000000000000896 32756262 10.1097/NNE.0000000000000896

[CR22] Cooper, C., Booth, A., Britten, N., & Garside, R. (2017). A comparison of results of empirical studies of supplementary search techniques and recommendations in review methodology handbooks: A methodological review. *Systematic Reviews,**6*(1), Article 234. 10.1186/s13643-017-0625-1 29179733 10.1186/s13643-017-0625-1 PMC5704629

[CR23] Devries, K. M., & Naker, D. (2021). Preventing teacher violence against children: The need for a research agenda. *The Lancet Global Health,**9*(4), e379–e380. 10.1016/S2214-109X(21)00093-0 33740400 10.1016/S2214-109X(21)00093-0

[CR24] Dwayne Simpson, D. (2009). Organizational readiness for stage-based dynamics of innovation implementation. *Research on Social Work Practice*, *19*(5), 541-551. 10.1177/1049731509335589

[CR25] Esan, O. T., Maswime, S., & Blaauw, D. (2022). Organisational and individual readiness for change to respectful maternity care practice and associated factors in Ibadan, Nigeria: A cross-sectional survey. *BMJ Open,**12*(11), Article Article e065517. 10.1136/bmjopen-2022-065517 36414287 10.1136/bmjopen-2022-065517 PMC9685001

[CR26] Faris, M. M., Shepherd, H. L., Butow, P. N., Kelly, P., He, S., Rankin, N., Masya, L., Group, A. P., & Shaw, J. (2023). Staff- and service-level factors associated with organisational readiness to implement a clinical pathway for the identification, assessment, and management of anxiety and depression in adults with cancer. *BMC Health Services Research,**23*(1), 866. 10.1186/s12913-023-09829-2 37582818 10.1186/s12913-023-09829-2 PMC10426102

[CR27] Fry, D., Fang, X., Elliott, S., Casey, T., Zheng, X., Li, J., Florian, L., & McCluskey, G. (2018). The relationships between violence in childhood and educational outcomes: A global systematic review and meta-analysis. *Child Abuse & Neglect,**75*, 6–28. 10.1016/j.chiabu.2017.06.021 28711191 10.1016/j.chiabu.2017.06.021

[CR28] Gagne, M. H., Drapeau, S., & Clement, M. E. (2020). Community readiness for child maltreatment prevention: The challenge of a brief assessment. *The Journal of Primary Prevention,**41*(4), 299–316. 10.1007/s10935-020-00591-3 32557219 10.1007/s10935-020-00591-3

[CR29] Gagnon, M. P., Attieh, R., el Ghandour, K., Legare, F., Ouimet, M., Estabrooks, C. A., & Grimshaw, J. (2014). A systematic review of instruments to assess organizational readiness for knowledge translation in health care. *PLoS One,**9*(12), Article e114338. 10.1371/journal.pone.0114338 25474622 10.1371/journal.pone.0114338 PMC4256226

[CR30] Galdames, S., Montecinos, C., Campos, F., Ahumada, L., & Leiva, M. V. (2017). Novice principals in Chile mobilizing change for the first time: Challenges and opportunities associated with a school’s readiness for change. *Educational Management Administration & Leadership,**46*(2), 318–338. 10.1177/1741143217707520

[CR31] Gist, M., & Mitchell, T. (1992). Self-efficacy: A theoretical analysis of its determinants and malleability. *Academy of Management Review,**17*(2), 183–211. 10.2307/258770

[CR32] Glanz, K., Rimer, B. K., & Viswanath, K. (2015). *Health Behavior : Theory, Research, and Practice* (K. Glanz, B. K. Rimer, & K. K. Viswanath, Eds. Fifth ed.).

[CR33] Grant, M. J., & Booth, A. (2009). A typology of reviews: An analysis of 14 review types and associated methodologies. *Health Information and Libraries JournAl,**26*(2), 91–108. 10.1111/j.1471-1842.2009.00848.x 19490148 10.1111/j.1471-1842.2009.00848.x

[CR34] Guo, S., Chen, J., Yu, B., Jiang, Y., Song, Y., & Jin, Y. (2019). Knowledge, attitude and practice of child sexual abuse prevention among parents of children with hearing loss: A pilot study in Beijing and Hebei province, China. *Journal of Child Sexual Abuse*, *28*(7), 781–798. 10.1080/10538712.2019.1627688 10.1080/10538712.2019.1627688 31287784

[CR35] Haddaway, N. R., Collins, A. M., Coughlin, D., & Kirk, S. (2015). The role of Google Scholar in evidence reviews and its applicability to grey literature searching. *PLoS One*, *10*(9), e0138237. 10.1371/journal.pone.0138237 10.1371/journal.pone.0138237 PMC4574933 26379270

[CR36] Hailes, H. P., Yu, R., Danese, A., & Fazel, S. (2019). Long-term outcomes of childhood sexual abuse: An umbrella review. *The Lancet Psychiatry,**6*(10), 830–839. 10.1016/S2215-0366(19)30286-X 31519507 10.1016/S2215-0366(19)30286-X PMC7015702

[CR37] Hannon, P. A., Garson, G., Harris, J. R., Hammerback, K., Sopher, C. J., & Clegg-Thorp, C. (2012). Workplace health promotion implementation, readiness, and capacity among midsize employers in low-wage industries: A national survey. *Journal of Occupational and Environmental Medicine,**54*(11), 1337–1343. 10.1097/JOM.0b013e3182717cf2 23090160 10.1097/JOM.0b013e3182717cf2 PMC3493879

[CR38] Harris, L. G., Higgins, D. J., Willis, M. L., Lawrence, D., Mathews, B., Thomas, H. J., Malacova, E., Pacella, R., Scott, J. G., Finkelhor, D., Meinck, F., Erskine, H. E., & Haslam, D. M. (2024). The prevalence and patterns of maltreatment, childhood adversity, and mental health disorders in an Australian out-of-home care sample. *Child Maltreatment*. 10.1177/10775595241246534 38627990 10.1177/10775595241246534 PMC11656622

[CR39] Helfrich, C. D., Li, Y.-F., Sharp, N. D., & Sales, A. E. (2009). Organizational readiness to change assessment (ORCA): Development of an instrument based on the Promoting Action on Research in Health Services (PARIHS) framework. *Implementation Science,**4*(1), 1–13. 10.1186/1748-5908-4-38 19594942 10.1186/1748-5908-4-38 PMC2716295

[CR40] Hjort, A. V., Schreuders, M., Rasmussen, K. H., & Klinker, C. D. (2021). Are Danish vocational schools ready to implement “smoke-free school hours”? A qualitative study informed by the theory of organizational readiness for change. *Implementation Science Communications,**2*(1), Article 40. 10.1186/s43058-021-00140-x 33836841 10.1186/s43058-021-00140-x PMC8033695

[CR41] Holt, D. T., Armenakis, A. A., Feild, H. S., & Harris, S. G. (2007b). Readiness for organizational change: The systematic development of a scale. *The Journal of Applied Behavioral Science,**43*(2), 232–255. 10.1177/0021886306295295

[CR42] Holt, D. T., Armenakis, A. A., Feild, H. S., & Harris, S. G. (2016). Readiness for organizational change. *The Journal of Applied Behavioral Science,**43*(2), 232–255. 10.1177/0021886306295295

[CR43] Holt, D. T., Helfrich, C. D., Hall, C. G., & Weiner, B. J. (2010). Are you ready? How health professionals can comprehensively conceptualize readiness for change. *Journal of General Internal Medicine,**25*(Suppl 1), 50–55. 10.1007/s11606-009-1112-8 20077152 10.1007/s11606-009-1112-8 PMC2806967

[CR44] Holt, D., Armenakis, A., Harris, S., & Feild, H. (2007a). Toward a comprehensive definition of readiness for change: A review of research and instrumentation. *Research in Organizational Change and Development,**16*, 289–336. 10.1016/S0897-3016(06)16009-7

[CR45] Hughes, K., Bellis, M. A., Hardcastle, K. A., Sethi, D., Butchart, A., Mikton, C., Jones, L., & Dunne, M. P. (2017). The effect of multiple adverse childhood experiences on health: A systematic review and meta-analysis. *Lancet Public Health,**2*(8), e356–e366. 10.1016/S2468-2667(17)30118-4 29253477 10.1016/S2468-2667(17)30118-4

[CR46] Hustus, C. L., & Owens, J. S. (2018). Assessing readiness for change among school professionals and its relationship with adoption and reported implementation of mental health initiatives. *Child & Youth Care Forum,**47*(6), 829–844. 10.1007/s10566-018-9463-0

[CR47] Ittner, D., Hagenauer, G., & Hascher, T. (2019). Swiss principals’ emotions, basic needs satisfaction and readiness for change during curriculum reform. *Journal of Educational Change,**20*(2), 165–192. 10.1007/s10833-019-09339-1

[CR48] Jippes, M., Driessen, E. W., Broers, N. J., Majoor, G. D., Gijselaers, W. H., & van der Vleuten, C. P. (2013). A medical school’s organizational readiness for curriculum change (MORC): Development and validation of a questionnaire. *Academic Medicine,**88*(9), 1346–1356. 10.1097/ACM.0b013e31829f0869 23887017 10.1097/ACM.0b013e31829f0869

[CR49] Jones, R. A., Jimmieson, N. L., & Griffiths, A. (2005). The Impact of organizational culture and reshaping capabilities on change implementation success: The mediating role of readiness for change. *Journal of Management Studies*, *42*(2), 361-386.10.1111/j.1467-6486.2005.00500.x

[CR50] Kondakçı, Y., & Zayim Kurtay, M. (2013). Development and validation of readiness for change scale. *Elementary Education Online,**12*(1), 23–25.

[CR51] Kostadinov, I., Daniel, M., Stanley, L., Gancia, A., & Cargo, M. (2015). A systematic review of community readiness tool applications: Implications for reporting. *International Journal Environmental Research Public Health,**12*(4), 3453–3468. 10.3390/ijerph120403453 10.3390/ijerph120403453 PMC4410196 25811769

[CR52] Lawrence, D. M., Hunt, A., Mathews, B., Haslam, D. M., Malacova, E., Dunne, M. P., Erskine, H. E., Higgins, D. J., Finkelhor, D., Pacella, R., Meinck, F., Thomas, H. J., & Scott, J. G. (2023). The association between child maltreatment and health risk behaviours and conditions throughout life in the Australian Child Maltreatment Study. *Medical Journal of Australia,**218*(Suppl 6), S34–S39. 10.5694/mja2.51877 37004181 10.5694/mja2.51877 PMC10952518

[CR53] Le, K., Chen, T. A., Martinez Leal, I., Correa-Fernandez, V., Obasi, E. M., Kyburz, B., Williams, T., Casey, K., Brown, H. A., O’Connor, D. P., & Reitzel, L. R. (2021). Organizational-level moderators impacting tobacco-related knowledge change after tobacco education training in substance use treatment centers. *International Journal of Environmental Research and Public Health*. 10.3390/ijerph18147597 34300052 10.3390/ijerph18147597 PMC8305177

[CR54] Lehman, W. E., Greener, J. M., & Simpson, D. D. (2002). Assessing organizational readiness for change. *Journal of Substance Abuse Treatment,**22*(4), 197–209. 12072164 10.1016/s0740-5472(02)00233-7

[CR55] Letourneau, E. J., Brown, D. S., Fang, X., Hassan, A., & Mercy, J. A. (2018). The economic burden of child sexual abuse in the United States. *Child Abuse & Neglect,**79*, 413–422. 10.1016/j.chiabu.2018.02.020 29533869 10.1016/j.chiabu.2018.02.020 PMC6542279

[CR56] Lewin, K. (1947). Frontiers in group dynamics: II. Channels of group life; social planning and action research. *Human Relations (New York),**1*(2), 143–153. 10.1177/001872674700100201

[CR57] Lidegaard, L. P., Kristiansen, M., & Pisinger, C. (2021). Readiness for implementation of smoke-free work hours in private companies: A qualitative study of perceptions among middle managers. *Tobacco Prevention & Cessation,**7*, 38. 10.18332/tpc/134800 34056145 10.18332/tpc/134800 PMC8145195

[CR58] Maniglio, R. (2013). Child sexual abuse in the etiology of anxiety disorders: A systematic review of reviews. *Trauma, Violence & Abuse,**14*(2), 96–112. 10.1177/1524838012470032 10.1177/1524838012470032 23262751

[CR59] Marson, B. (2024). The impact of childhood sexual abuse on interpersonal relationships: A cross-sectional study in Trinidad [Report]. *Journal of International Women’s Studies*, *26*, COV13+. https://link.gale.com/apps/doc/A798419424/AONE?u=qut&sid=bookmark-AONE&xid=837e13c6

[CR60] McCarthy-Jones, S., & McCarthy-Jones, R. (2014). Body mass index and anxiety/depression as mediators of the effects of child sexual and physical abuse on physical health disorders in women. *Child Abuse & Neglect,**38*(12), 2007–2020. 10.1016/j.chiabu.2014.10.012 25459987 10.1016/j.chiabu.2014.10.012

[CR61] Mikton, C., Mehra, R., Butchart, A., Addiss, D., Almuneef, M., Cardia, N., Cheah, I., Chen, J., Makoae, M., & Raleva, M. (2011). A multidimensional model for child maltreatment prevention readiness in low- and middle-income countries. *Journal of Community Psychology,**39*(7), 826–843. 10.1002/jcop.20474

[CR62] Mikton, C., Power, M., Raleva, M., Makoae, M., Al Eissa, M., Cheah, I., Cardia, N., Choo, C., & Almuneef, M. (2013). The assessment of the readiness of five countries to implement child maltreatment prevention programs on a large scale. *Child Abuse and Neglect*, *37*(12), 1237-1251.10.1016/j.chiabu.2013.07.009 10.1016/j.chiabu.2013.07.009 23962585

[CR63] Nickerson, A., Kim, S., Dudley, M., Livingston, J. A., & Manges, M. (2021). Longitudinal impact of the Second Step Child Protection Unit on teacher knowledge, attitude, and climate. *Children and Youth Services Review*. 10.1016/j.childyouth.2020.105892

[CR64] Octaria, Y., Apriningsih, A., Dwiriani, C. M., & Februhartanty, J. (2021). School readiness to adopt a school-based adolescent nutrition intervention in urban Indonesia. *Public Health Nutrition,**24*(S2), s72–s83. 10.1017/S1368980020001299 32375906 10.1017/S1368980020001299 PMC10071219

[CR65] Oetting, E. R., Donnermeyer, J. F., Plested, B. A., Edwards, R. W., Kelly, K., & Beauvais, F. (1995). Assessing community readiness for prevention. *International Journal of the Addictions,**30*(6), 659–683. 10.3109/10826089509048752 7657396 10.3109/10826089509048752

[CR66] Oostendorp, L. J., Durand, M. A., Lloyd, A., & Elwyn, G. (2015). Measuring organisational readiness for patient engagement (MORE): An international online delphi consensus study. *BMC Health Services Research,**15*, Article 61. 10.1186/s12913-015-0717-3 25879457 10.1186/s12913-015-0717-3 PMC4334597

[CR67] Orchowski, L. M., Oesterle, D. W., Zong, Z. Y., Bogen, K. W., Elwy, A. R., Berkowitz, A. D., & Pearlman, D. N. (2023). Implementing school-wide sexual assault prevention in middle schools: A qualitative analysis of school stakeholder perspectives. *Journal of Community Psychology,**51*(3), 1314–1334. 10.1002/jcop.22974 36468237 10.1002/jcop.22974

[CR68] Payne, A. A., & Eckert, R. (2010). The relative importance of provider, program, school, and community predictors of the implementation quality of school-based prevention programs. *Prevention Science,**11*(2), 126–141. 10.1007/s11121-009-0157-6 19902357 10.1007/s11121-009-0157-6

[CR69] Plested, B., Smitham, D. M., Jumper-Thurman, P., Oetting, E. R., & Edwards, R. W. (1999). Readiness for drug use prevention in rural minority communities. *Substance Use & Misuse,**34*(4–5), 521–544. 10.3109/10826089909037229 10210091 10.3109/10826089909037229

[CR70] Prochaska, J. O. (1994). *The transtheoretical approach: Crossing traditional boundaries of therapy*. Krieger Pub.

[CR71] Proctor, E. K., Powell, B. J., Baumann, A. A., Hamilton, A. M., & Santens, R. L. (2012). Writing implementation research grant proposals: Ten key ingredients. *Implementation Science,**7*(1), Article 96. 10.1186/1748-5908-7-96 23062065 10.1186/1748-5908-7-96 PMC3541090

[CR72] Rethlefsen, M. L., Kirtley, S., Waffenschmidt, S., Ayala, A. P., Moher, D., Page, M. J., Koffel, J. B., Group, P.-S. (2021). PRISMA-S: An extension to the PRISMA statement for reporting literature searches in systematic reviews. *Systematic Reviews,**10*(1), Article 39. 10.1186/s13643-020-01542-z 34285662 10.5195/jmla.2021.962 PMC8270366

[CR73] Rogers, E. M. (1983). *Diffusion of innovations (3rd ed.)*. Free Press.

[CR74] Rojewski, A. M., Bailey, S. R., Bernstein, S. L., Cooperman, N. A., Gritz, E. R., Karam-Hage, M. A., Piper, M. E., Rigotti, N. A., & Warren, G. W. (2019). Considering systemic barriers to treating tobacco use in clinical settings in the United States. *Nicotine & Tobacco Research,**21*(11), 1453–1461. 10.1093/ntr/nty123 29917118 10.1093/ntr/nty123 PMC6941704

[CR75] Russell, D., Higgins, D., & Posso, A. (2020). Preventing child sexual abuse: A systematic review of interventions and their efficacy in developing countries. *Child Abuse & Neglect,**102*, Article 104395. 10.1016/j.chiabu.2020.104395 32062425 10.1016/j.chiabu.2020.104395

[CR76] Scaccia, J. P., Cook, B. S., Lamont, A., Wandersman, A., Castellow, J., Katz, J., & Beidas, R. S. (2015). A practical implementation science heuristic for organizational readiness: R = MC(2). *Journal of Community Psychology,**43*(4), 484–501. 10.1002/jcop.21698 26668443 10.1002/jcop.21698 PMC4676714

[CR77] Sharma, S. V., Upadhyaya, M., Schober, D. J., & Byrd-Williams, C. (2014). A conceptual framework for organizational readiness to implement nutrition and physical activity programs in early childhood education settings. *Preventing Chronic Disease,**11*, E190. 10.5888/pcd11.140166 25357258 10.5888/pcd11.140166 PMC4215571

[CR78] Shea, C. M., Jacobs, S. R., Esserman, D. A., Bruce, K., & Weiner, B. J. (2014). Organizational readiness for implementing change: A psychometric assessment of a new measure. *Implementation Science,**9*(1), Article 7. 10.1186/1748-5908-9-7 24410955 10.1186/1748-5908-9-7 PMC3904699

[CR79] Shipe, S. L., Guastaferro, K., Noll, J. G., Connell, C. M., Morgan, P. L., & Crowley, D. M. (2022). Taking a school-based child sexual abuse prevention program to scale: A cost analysis. *Prevention Science,**23*(8), 1394–1403. 10.1007/s11121-022-01401-4 35867317 10.1007/s11121-022-01401-4 PMC11318369

[CR80] Stallones, L., Gibbs-Long, J., Gabella, B., & Kakefuda, I. (2008). Community readiness and prevention of traumatic brain injury. *Brain Injury,**22*(7–8), 555–564. 10.1080/02699050802132487 18568708 10.1080/02699050802132487

[CR81] Swahn, M. H., Robow, Z., Umenze, F., Balenger, A., Dumbili, E. W., & Obot, I. (2022). A readiness assessment for the prevention of alcohol-related harm in West Africa: A new methodological approach to inform practice and policy. *International Journal of Drug Policy, 103*, 103650–103650. 10.1016/j.drugpo.2022.103650 35339092

[CR82] Swindle, T., Whiteside-Mansell, L., & Johnson, D. (2018). Organizational readiness for implementing a nutrition curriculum in early care and education. *Journal of Nutrition Education and Behavior,**50*(7), S78. 10.1016/j.jneb.2018.04.129

[CR83] Thaker, S., Steckler, A., Sanchez, V., Khatapoush, S., Rose, J., & Hallfors, D. D. (2008). Program characteristics and organizational factors affecting the implementation of a school-based indicated prevention program. *Health Education Research,**23*(2), 238–248. 10.1093/her/cym025 17639122 10.1093/her/cym025

[CR84] Thurman, P. J., Vernon, I. S., & Plested, B. (2007). Advancing HIV/AIDS prevention among American Indians through capacity building and the community readiness model. *Journal of Public Health Management and Practice,**13*(Supplement), S49–S54. 10.1097/00124784-200701001-00009 10.1097/00124784-200701001-00009 17159467

[CR85] Turell, S., Herrmann, M., Hollander, G., & Galletly, C. (2012). Lesbian, gay, bisexual, and transgender communities’ readiness for intimate partner violence prevention. *Journal of Gay & Lesbian Social Services,**24*(3), 289–310. 10.1080/10538720.2012.697797

[CR86] Ullman, S. E. (2016). Sexual revictimization, PTSD, and problem drinking in sexual assault survivors. *Addictive Behaviors,**53*, 7–10. 10.1016/j.addbeh.2015.09.010 26414205 10.1016/j.addbeh.2015.09.010 PMC4679471

[CR87] Walsh, K., Berthelsen, D., Nicholson, J. M., Brandon, L., Stevens, J., & Rachele, J. N. (2013). Child sexual abuse prevention education: A review of school policy and curriculum provision in Australia. *Oxford Review of Education,**39*(5), 649–680. 10.1080/03054985.2013.843446

[CR88] Weiner, B. J. (2009). A theory of organizational readiness for change. *Implementation Science,**4*(1), Article 67. 10.1186/1748-5908-4-67 19840381 10.1186/1748-5908-4-67 PMC2770024

[CR89] Weiner, B. J., Amick, H., & Lee, S. Y. (2008). Conceptualization and measurement of organizational readiness for change: A review of the literature in health services research and other fields. *Medical Care Research and Review,**65*(4), 379–436. 10.1177/1077558708317802 18511812 10.1177/1077558708317802

[CR90] Weiner, B. J., Clary, A. S., Klaman, S. L., Turner, K., & Alishahi-Tabriz, A. (2020). Organizational readiness for change: What we know, what we think we know, and what we need to know. In B. Albers, A. Shlonsky, & R. Mildon (Eds.), *Implementation Science 3.0* (pp. 101–144). Springer International Publishing. 10.1007/978-3-030-03874-8_5

[CR91] Winters, A. M., Collins-Camargo, C., Antle, B. F., & Verbist, A. N. (2020). Implementation of system-wide change in child welfare and behavioral health: The role of capacity, collaboration, and readiness for change. *Children and Youth Services Review*. 10.1016/j.childyouth.2019.10458033814658

[CR92] World Health Organization. (2006). Preventing child maltreatment: A guide to taking actions and generating evidence. *Geneva*, 10.

[CR93] World Health Organization. (2013). *Readiness Assessment for the Prevention of Child Maltreatment (RAP-CM)*. https://www.who.int/violence_injury_prevention/violence/child/cmp_readiness/en/

[CR94] World Health Organization. (2018). *INSPIRE Handbook : Action for Implementing the Seven Strategies for Ending Violence Against Children*. World Health Organization.

[CR95] World Health Organization. (2019). *School-Based Violence Prevention: A Practical Handbook*. World Health Organization. https://apps.who.int/iris/handle/10665/324930

[CR96] Zakaria, Z., & Ismail, S. N. (2020). The relationship between organizational readiness to change and professional learning community (PLC) practices in Kelantan residential school. *International Journal of Management and Humanities,**4*(6), 73–77. 10.35940/ijmh.F0611.024620

[CR97] Zhang, W., Chen, J., & Liu, F. (2015). Preventing child sexual abuse early: Preschool teachers’ knowledge, attitudes, and their training education in China. *SAGE Open*, *5*(1), 1-8. 10.1177/2158244015571187

